# The effects of 2 % inclusion of blue mussel (*Mytilus edulis*) meal on the performance and clinical welfare of fast-growing broiler chickens

**DOI:** 10.1016/j.psj.2026.107153

**Published:** 2026-05-20

**Authors:** Fernanda Machado Tahamtani, Hilde Faaland Schøyen, Käthe Kittelsen

**Affiliations:** a Animalia – Norwegian Meat and Poultry Research Centre, 0585 Oslo, Norway; b Fiskå Mølle AS, Stasjonsveien 1640 Råde, Norway

**Keywords:** Blue mussel meal, Broiler nutrition, Alternative protein sources, Electrolyte homeostasis, Animal welfare

## Abstract

Sustainable feed ingredients are increasingly needed to support environmentally responsible poultry production, particularly those that reduce dependence on conventional protein sources such as soybean meal and fishmeal. Blue mussel meal (BMM) offers potential as a locally produced, circular-economy protein ingredient; however, high and variable mineral content—especially sodium and chloride—has raised concerns regarding its safe dietary inclusion. This study therefore evaluated the effects of a nutritionally balanced diet containing 2 % BMM on broiler chicken performance, physiology, and welfare. A total of 216 Ross 308 broilers were assigned to either a commercial control feed or a diet including 2 % BMM formulated to match the control diet in sodium and chloride content. Live weight, feed intake, water intake, and water‑to‑feed ratio were measured throughout the rearing period. Plasma sodium, chloride, creatinine, and urea were assessed at three ages (13, 21, and 29 days). At 32 days of age, all birds underwent a clinical welfare assessment including gait score, footpad dermatitis, hock burn, cleanliness, vent pasting, and leg deformities. Live weight and feed intake did not differ between treatments (*P* > 0.20). Water intake showed an interaction between diet and age (*P* = 0.005), with BMM-fed birds consuming 25.6 ml more water per bird by day 32. Water‑to‑feed ratio tended to be higher in the BMM group (*P* = 0.051). Plasma sodium and chloride concentrations did not differ between diets, and no elevations in creatinine or urea were observed. Welfare outcomes were comparable between groups, with a tendency for improved gait in BMM-fed birds (*P* = 0.08) and no cases of footpad dermatitis in either treatment. Overall, the inclusion of 2 % BMM—when formulated to appropriate sodium and chloride levels—did not adversely affect growth, hydration physiology, or welfare. However, further studies must assess these parameters also under full-scale production.

## Introduction

Global food production is increasingly shaped by demands for sustainable resource use, reduced environmental impact, and resilience in the face of climate change, as highlighted, for example, in the European Green Deal and Farm to Fork strategy (European Commission, 2019, 2020). In livestock and poultry industries, the search for alternative feed ingredients that reduce reliance on conventional protein sources—particularly soybean meal and fishmeal—has received considerable attention. Sustainable feed strategies are central to broader “green transition” initiatives, which emphasize circular value chains, reduced waste, and local resource utilization. Blue mussels (*Mytilus spp*.) have emerged as a promising candidate in this context (Suplicy, 2020). They require no fertilization or feed inputs, help improve water quality through filter-feeding, and can be cultivated along many coastlines (Kotta et al., 2020).

Research evaluating the nutritional and functional properties of blue mussel meal (**BMM**) in poultry diets is still limited but expanding. The amino acid composition and digestibility of BMM has been found comparable to that of fish meal and suitable for monogastric nutrition (Ahlstrøm et al., 2026). In broilers, a study has reported acceptable growth performance, feed conversion ratio, feed intake and mortality in broilers fed a diet with 12 % inclusion of blue mussels replacing fish meal (Waldenstedt and Jönsson, 2006). Work in laying hens has focused primarily on egg quality and yolk pigmentation, with findings showing that mussel-derived carotenoids can influence yolk colour and that BMM may partially replace conventional protein sources without compromising egg production or egg quality under controlled dietary formulations (Afrose et al., 2016; Jönsson et al., 2011; van der Heide et al., 2021a). However, the chemical composition of mussel meal—particularly in salt content—remains a major challenge in formulating predictable and safe diets (Tahamtani et al., 2026). Important knowledge gaps persist regarding how different processing methods, inclusion levels, and nutrient interactions influence both physiological responses and welfare outcomes in poultry. In a recent study, Tahamtani et al. (2026) underscored these concerns by documenting that mussel meal can contain very high sodium and chloride concentrations and, if not balanced correctly, may result in salt intoxication and associated welfare impairments in broiler chickens, including high mortality. Furthermore, most studies have investigated the inclusion of blue mussels as an alternative to fish meal, but few have assessed the replacement of soy protein.

The aim of this study was, therefore, to assess the effects of the provision of a diet containing a low inclusion rate of BMM on the growth performance and welfare of broiler chickens. We hypothesised that, as the levels of salt in the feed containing BMM were carefully considered, there would be no difference between the two groups in their feed and water intake, their weight and growth curve, welfare parameters such as footpad dermatitis, gait score, cleanliness, vent pasting and hock burns or physiological blood parameters. This study was part of a larger study, comparing the effects of a diet with 2 % BMM on broiler chicken meat quality parameters (Münch et al., in prep).

## Materials and methods

### Ethical statement

All procedures involving animals were approved by the Norwegian Food Safety Authorities, ID number 31396.

### Animals, diets and experimental design

This study used 231 Ross 308 broiler chickens which were incubated and hatched at a commercial hatchery and transported to the experimental facility as 0 days of age. Upon arrival at the experimental facilities, the birds were randomly distributed into 12 pens (6 pens per diet treatment) located in the same room each measuring 296 × 60 × 71 cm (length × height × depth), with 18 birds per pen. The 15 extra chickens were placed in an identical “reserve” pen in the same room. Each pen was equipped with 1 round feeder (34 × 40 cm height × width) and 1 bell drinker (30 × 32 cm height × width). Water and feed were available ad libitum. Wood shavings were used as litter material and environmental enrichment was provided in every pen as a litter tray (50 × 35 × 12 cm, length × width × height) filled with peat for dust bathing. Starting with continuous light on the day of arrival, the light was gradually adjusted to a schedule of 18 h light: 6 h dark by one week of age. The temperature in the room started at approx. 34°C on day 1 and gradually decreased thereafter until reaching 18°C on day 32 of age. Relative humidity ranged from 52 to 65 %.

From day 0 to day 7 of age, all pens received a commercial starter feed for broiler chickens and the birds were allowed to habituate to their environment. At the beginning of day 8, to ensure a group size of 18 birds per pen at the start of the test period, the numbers of birds in the 12 main pens were supplemented from the 15 extra birds in the reserve pen. At this point, the reserve pen was dismantled and any extra birds beyond the 216 individuals included in the study were humanely euthanised by blunt force trauma to the head followed by cervical dislocation.

The experiment employed a controlled, two‑treatment design consisting of a Control group and the Blue mussel group with 6 pens randomly assigned to each group. The birds in the Control group transitioned from the starter feed to a standard commercial grower feed for broiler chickens. The Blue Mussel group were transitioned from the starter feed to a specially designed feed which included 2 % of BMM. The blue mussels were harvested from production facilities in Denmark where they are mainly grown as an ecosystem service to remedy the eutrophication of local waters. The mussels were steamed, and the shells were separated from the soft tissue, which was subsequently dried into a meal. Table 1 presents the nutritional composition of the BMM. Table 2, Table 3 present the nutritional composition and the macronutrients of the diet treatments, respectively. The birds received these diets from day 8 until the end of the study at 33 days of age, at which point the birds were sent to slaughter at commercial slaughterhouse. All diets were produced in pelleted form (ø: starter feed = 2.5 mm, Control and Blue Mussel feed = 3 mm).

**Table 1:** Nutritional composition of blue mussel meal (g/kg and mg/kg meal).

Dry blue mussel meal	g/kg
Dry matter	929.0
Crude protein	467.0
Crude fat	30.0
Crude ash	256.0
Nitrogen-free Extract*	176.0
Calcium	55.0
Phosphorus	6.9
Sodium	38.0
Chloride	66.9
Potassium	13.0
Magnesium	5.8
	**mg/kg**
Zink	70.0
Iron	1500.0
Manganese	180.0
Copper	8.7
Iodine	55.0
Selenium	3.7
Cobalt	1.4
Molybdenum	1.1
Arsenic	7.8
Lead	1.0
Cadmium	0.35
Mercury	0.03

⁎ Calculated by difference. Blue mussel meal was analyzed prior to formulation for crude protein (Method 990.03, AOAC, 2006), moisture (Method 934.01, AOAC, 2006), crude fat (Method 920.39 [A], AOAC), crude fiber (Method 978.10, AOAC, 2006), and ash (Method 942.05, AOAC, 2006) at LabTek laboratory at Norwegian University of Life Sciences (Ås, Norway). Mineral analysis of blue mussel meal was performed at Eurofins (Moss, Norway).

**Table 2:** Composition of the diets (g/kg feed).

Items	Starter diet (day 0-7)	Grower diets (day 7-33)	
		Control	Blue Mussel
**Ingredient**			
Blue mussel meal	-	-	21
Soybean meal	198	144	130
Faba beans	10	30	30
Rapeseed cake expeller	-	50	50
Corn gluten	48.2	-	-
Wheat	231	480	500
Barley	-	-	8
Oats	100	170	137
Maize	174.3	-	-
Potato Protein	4	-	-
Peas	150	60	64
Soybean oil	40	10	13
Animal fat	-	27.4	23.7
Limestone	13.9	7.6	4.2
NaCl	0.8	0.5	-
Sodium diformate	2.0	1.0	-
Monocalcium phosphate	11.0	4.6	4.4
L-lysine HCL	3.6	3.2	2.9
DL-methionine	2.8	2.0	2.0
L-treonine	1.5	1.0	1.0
L-Valine	0.2	0.2	0.3
Tryptophane	0.3	-	-
Arginine	0.4	0.5	0.5
Vitamin and trace mineral mix 1	8	8	8
**Calculated content**			
Mussel meal	-	-	20
Metabolizable energy, MJ/kg 2	12.0	11.7	11.7
DEB (mEq/kg) 3	250	215	215

1 Vitamin- and mineral premix provided per kilogram of diet: Mn (from MnSO4⋅H2O), 65 mg; Zn (from ZnO), 55 mg; Fe (from FeSO4⋅7H2O), 50 mg; Cu (from CuSO4⋅5H2O), 8 mg; I [from Ca (IO3)2⋅H2O], 1.8 mg; Se, 0.30 mg; Co (from Co2O3), 0.20 mg; Mo, 0.16 mg. 2 Vitamin premix provided per kilogram of diet: vitamin A (from vitamin A acetate), 11,500 IU; cholecalciferol, 2,100 IU; vitamin E (from dl-α-tocopheryl acetate), 22 IU; vitamin B12, 0.60 mg; riboflavin, 4.4 mg; nicotinamide, 40 mg; calcium pantothenate, 35 mg; menadione (from menadione dimethylpyrimidinol), 1.50 mg; folic acid, 0.80 mg; thiamine, 3 mg; pyridoxine, 10 mg; biotin, 1 mg; choline chloride, 560 mg; ethoxyquin, 125 mg.

2 Calculated metabolizable energy poultry based on the CVB Feed Table (CVB, 2022).

3 Electrolyte Balance: represents dietary Na +K − Cl in mEq/kg of diet.

**Table 3:** Nutritional composition of diets (g/kg feed).

Items	Starter diet (day 0-7)		Grower diets (day 7-33)			
			Control		Blue mussel	
Content	Estimated	Analysed	Estimated	Analysed	Estimated	Analysed
Dry matter	892	889	889	883	889	883
Crude ash	58	56	48	44	47	44
Crude fat	70	61	80	60	80	55
Crude protein	219	231	198	181	198	188
Ca	8.9	9.2	6.4	6.1	5.9	6.7
K	8.0	7.8	7.2	7.6	7.3	7.1
P	6.1	6.1	5.0	5.3	5.1	5.2
Cl	1.8	1.8	2.2	1.7	2.7	2.4
Na	1.6	1.6	1.6	1.4	1.7	1.9
Mg	1.4	1.7	1.5	1.6	1.6	1.7
Zn (mg)	144	143	148	150	149	147
Fe (mg)	107	210	106	110	133	128
Cu (mg)	21	19	21	20	21	20
I (mg)	1.4	1.2	1.4	1.6	2.5	2.2
Se (mg)	0.6	0.5	0.6	0.6	0.6	0.6

### Data collection

The weight of the chickens was measured on day 0, 7, 22 and 33 of age and averaged per pen. Feed and water consumption data were collected daily by comparing the weight of the feed and water containers which were measured every day at 08:00. On day 13, 21 and 29 of age, one bird per pen was randomly selected and euthanised for the purpose of blood collection. The bird was removed from the pen, carried to an adjacent dissection room, stunned by blunt force trauma to the head and decapitated. Blood was then collected (approx. 2 ml) into plain blood collection tubes without additives for biochemistry and transported to the Central Laboratory at the Norwegian University of Life Sciences within 2 hours. These samples were used to monitor plasma concentrations of sodium (Na), chloride (Cl), urea and creatinine.

Twice per week, all birds were assessed by a veterinarian, who performed a visual screening for clinical signs of salt poisoning, such as laboured breathing, swelling of the head, poor growth, rejection of feed, wet litter and huddling (Gornatti-Churria et al., 2024; Klasing and Korver, 2020; Tahamtani et al., 2026). In case of doubt or birds showing signs of weakness, a hands-on clinical examination was performed in addition to the visual screening. Necropsy was conducted on all birds which died during the study, to establish tentative cause of death. The necropsy assessment followed a standard postmortem technique, in addition to a specific emphasis on water retention and kidney pathology.

On day 32 of age, a clinical welfare assessment was performed on all birds, including the assessment of gait score and cleanliness as well as the presence of footpad dermatitis, hock burns, vent pasting, valgus/varus leg deformities and bumblefoot. The birds were assessed by one singular experienced observer. Footpad dermatitis was scored on a three-point scale from 0 or no injury to 2 or serious injury (Ekstrand et al., 1997). Hock burns were scored on a four-point scale from 0 (no injury) to 3 (heavy crust formation on >10 % of the hock) (Sherlock et al., 2010). The incidence of bumblefoot (i.e., severe inflammatory state in the subcutaneous tissue causing a bulbous swelling of the footpad) (Welfare Quality, 2009) and vent pasting (i.e., excreta adhering to the plumage around the cloaca) were each scored on a dichotomous scale (yes/no). Plumage dirtiness was scored for the ventral part of the body on a four-point scale from 0 (very clean) to 3 (very dirty) according to the Welfare Quality assessment protocol for poultry (Welfare Quality, 2009). Finally, the incidence of varus/valgus deformities of the legs were defined as an inward or outward rotation of the tibiotarsus along the long axis of the bone (Bradshaw et al., 2002).

### Statistical analysis

Statistical analyses were conducted using SAS 9.4 (SAS Institute Inc., Cary NC). The data on the live bird weight, feed and water consumption as well as water to feed ratio were analysed with the mixed procedure with treatment and age (repeated measures) as fixed factors, as well as their interaction, and pen number as a random factor. Likewise, the same model was used to analyse the blood plasma concentrations of chloride and sodium. All data were checked for normality assumptions (i.e. independence and normal distribution of the residuals). Post-hoc analysis was performed with the Tukey test (Tukey’s HSD test).

The comparison of the total mortality between the two diet treatments was performed with the Student’s t test. The data on gait score, footpad dermatitis, hock burns, and cleanliness score were analysed using a multinomial distribution glimmix procedure with treatment as the fixed effect and the pen number as a random factor. The effect of the diet treatment on the incidence of vent pasting, valgus/varus leg deformities, and bumblefoot was examined using a binary glimmix procedure with a link logit distribution, also with treatment as a fixed factor and pen number as a random factor.

## Results

### Live weight

**Fig. 1:**
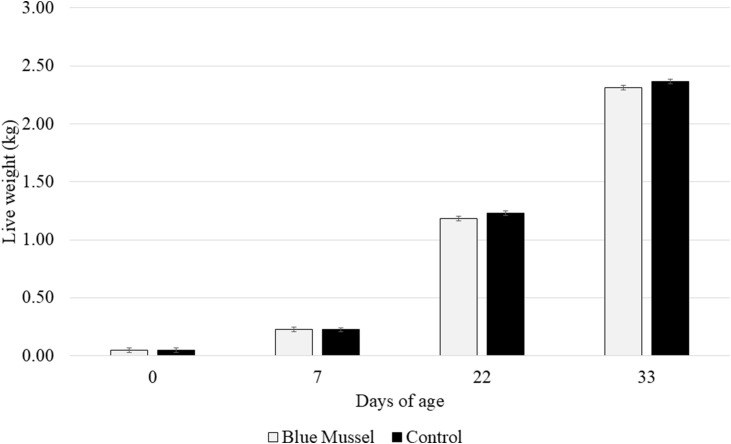
Live broiler chicken weight (kg, LS means ± SE) across diet treatments (Blue Mussel *n* = 6 pens; Control *n* = 6 pens) and age.

There was no effect of treatment (F_1,__10_ = 1.75; *P* = 0.21) or the interaction between treatment and age (F_3,__30_ = 1.43; *P* = 0.25) on the live weight of the birds. The only significant effect found was the expected effect of age (F_3,__30_ = 7875; *P* < 0.0001), with the birds gaining weight with age (Fig. 1).

### Feed and water intake

**Fig. 2:**
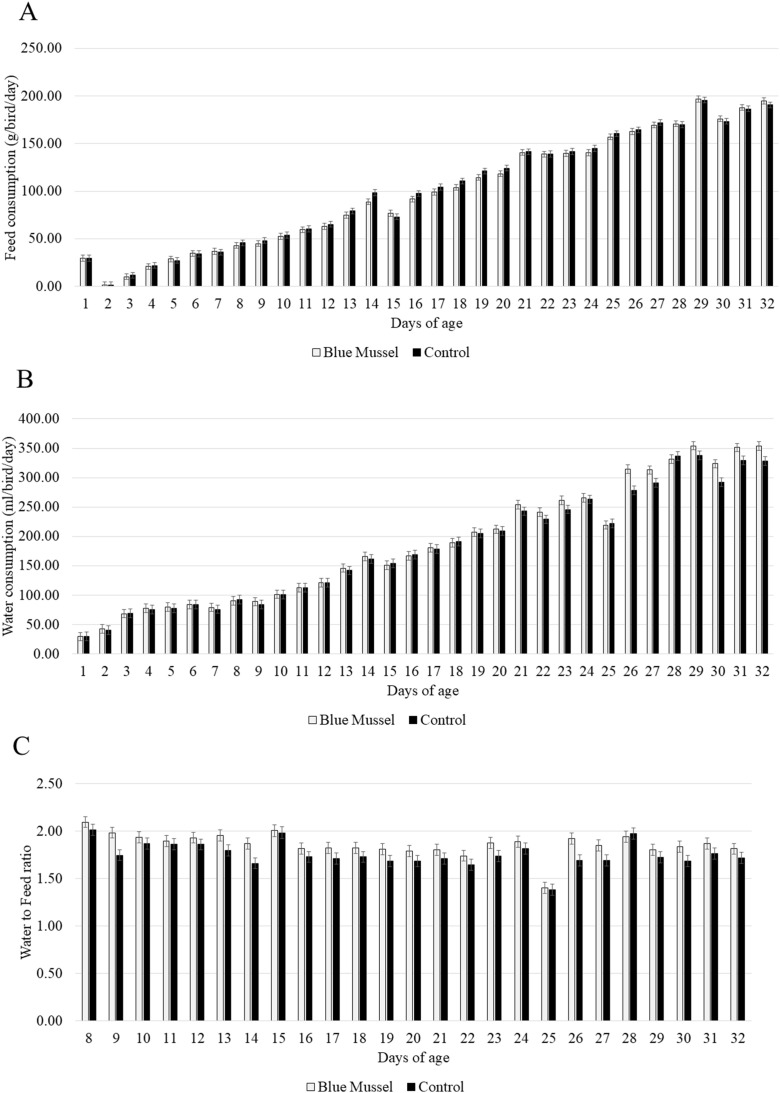
Daily consumption of feed (panel A), water (panel B), and water to feed ratio (panel C) across diet treatments (Blue Mussel *n* = 6 pens; Control *n* = 6 pens).

Feed intake was not affected by diet treatment (F_1,__41_ = 0.70; *P* = 0.40) or the interaction between feed and age (F_1,__370_ = 0.05; *P* = 0.82). The only observed effect on feed intake was age, with more feed being consumed per day as the birds grew older (F_1,__370_ = 11916.0; *P* < 0.0001, Fig. 2A).

Water consumption was affected by the interaction between diet treatment and age (F_1,__370_ = 8.13; *P* = 0.005). As can be seen in Fig. 2B, the two groups started to diverge around 21 days of age, with some fluctuation in the following days, and with the birds fed the Blue Mussel diet consuming on average 25.6 ml more water per bird compared to the Control birds by the end of the study at 32 days of age.

There was an effect of diet treatment on the water to feed consumption ratio (F_1,__165_ = 3.85; *P* = 0.051), with the birds in the blue mussel group presenting a higher water to feed ratio (LS Means ± SE: 1.86 ± 0.02) compared to the birds in the control group (LS Means ± SE: 1.75 ± 0.02; Fig. 2C).

### Clinical health, necropsy and mortality

All birds were assessed visually by veterinarians twice per week during the trial. Overall, the birds were alert in all pens, uniform in size and had a normal activity level as expected at different stages of the production period. No clinical signs of salt poisoning, as described by Gornatti-Churria et al. (2024), were observed. All birds that died or were culled during the trial (*n* = 11) were subjected to necropsy, performed by a poultry specialist. Causes for culling were primarily small, weak or lame birds. The postmortem examinations showed no signs of salt poisoning, like edema, swollen heads, wet carcasses, fluid in air sacs, swollen testis and kidneys (Gornatti-Churria et al., 2024; Klasing and Korver, 2020). The main findings were sudden death syndrome (Blue Mussel = 1; Control = 2), femoral head necrosis (Blue Mussel = 1; Control = 1), gizzard compaction (Blue Mussel = 1; Control = 0), and ascites (Blue Mussel = 1; Control = 1).

At the end of the experiment, at 33 weeks of age, there was no significant effect of diet treatment on mortality (t = −1.10; *P* = 0.29), with Control pens having an average final mortality of 3.67 ± 0.67 birds (20.4 %) and Blue Mussel pens having an average final mortality of 4.17 ± 0.57 birds (23.2 %).

### Plasma sodium and chloride levels

**Fig. 3:**
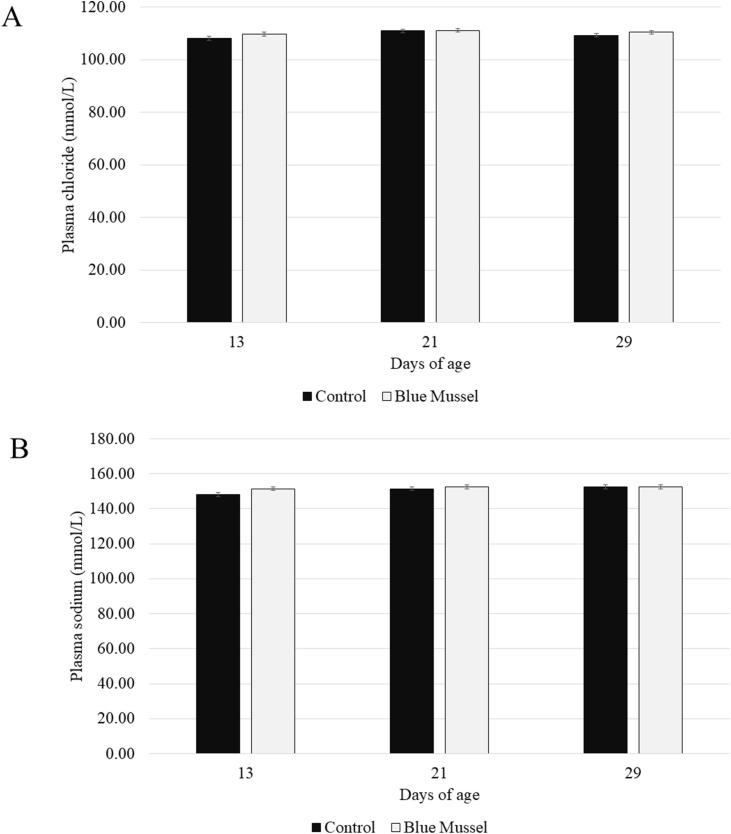
Concentration (mmol/L, LS means ± SE) of plasma chloride (panel A) and sodium (panel B) at 13, 21 and 29 days of age (Blue Mussel *n* = 6 birds/age; Control *n* = 6 birds/age).

There was no effect of treatment (F_1,__10_ = 1.89; *P* = 0.19) or the interaction between treatment and age on the concentration of plasma chloride (F_2,__20_ = 0.77; *P* = 0.48; Fig. 3A). There was however an effect of age (F_2,__20_ = 5.73; *P* = 0.01) with a higher concentration detected at 21 days of age compared to day 13 of age (*P* = 0.008; LS means ± SE: 111.08 mmol/*L* ± 0.51 and 109.00 mmol/*L* ± 0.51, respectively). There was no difference observed between day 29 (LS means ± SE: 109.92 mmol/*L* ± 0.51) of age and the other age points (*P* > 0.5).

Likewise, there was no effect of treatment (F_1,__10_ = 1.62; *P* = 0.23) or the interaction between treatment and age (F_2,__20_ = 1.51; *P* = 0.24; Fig. 3B) on the plasma concentration of sodium. There was an effect of age (F_2,__20_ = 4.16; *P* = 0.03) with a higher concentration at 29 days of age, compared to 13 days of age (*P* = 0.03; LS means ± SE: 152.58 mmol/*L* ± 0.81 and 149.83 mmol/*L* ± 0.81, respectively). There was also a tendency for the concentration at 21 days of age to be higher than at 13 days of age (*P* = 0.09; LS means ± SE: 152.08 mmol/*L* ± 0.81).

Regarding the concentration of creatinine, the values were all under the detection threshold of 13 µmol/L, except for one sample from a control bird at 13 days of age which had a concentration of 22 µmol/L. Likewise, the values for the concentration of urea were under the detection threshold of 1.80 mmol/L in all blood samples assessed.

### Welfare assessment

**Fig. 4:**
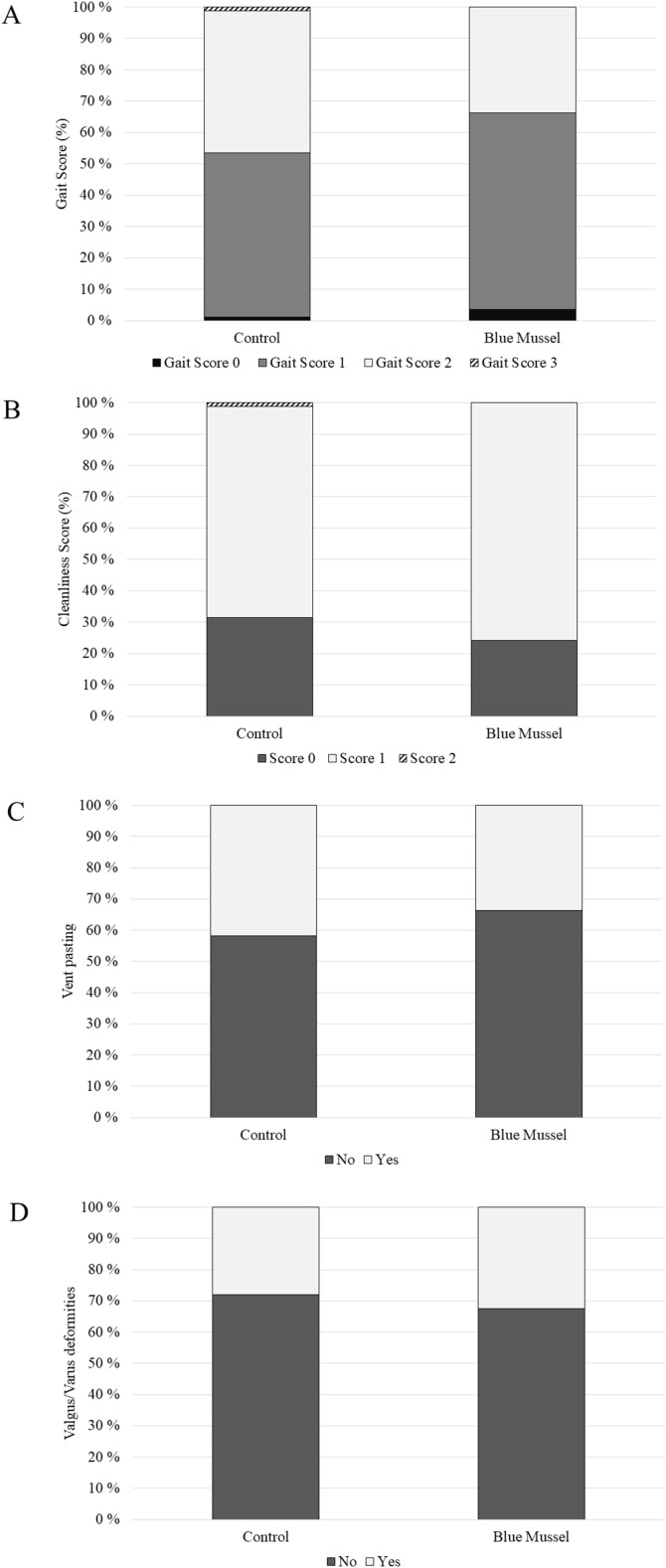
Frequency (%) of gait score (panel A), plumage cleanliness (panel B), vent pasting (panel C) and valgus/varus leg deformities (panel D) observed at 32 days of age. Higher scores represent higher severity of the welfare indicators. Blue mussel *n* = 83; Control *n* = 86.

There was a tendency for the birds fed the blue mussel feed to have better gait (i.e. be less lame) compared to the birds in the control group (F_1,__155_ = 3.16; *P* = 0.08; Fig. 4A). There was no effect of feed on the cleanliness scores (F_1,__156_ = 0.85; *P* = 0.35, Fig. 4B). There was also no effect of feed on the incidence of vent pasting (F_1,__157_ = 0.98; *P* = 0.32; Fig. 4C) or on the incidence of valgus/varus leg deformities (F_1,__157_ = 0.30; *P* = 0.58; Fig. 4D).

No birds presented with footpad dermatitis (i.e.100 % score 0) and no bumble feet were observed. Wounds were only observed in 4 birds, all in the control group. These were very small pecking wounds on the comb. All birds had hock burn score 0 except for two birds which presented hock burns score 1, both of which were in the group fed the blue mussel feed.

## Discussion

This study evaluated the effects of including 2 % BMM in broiler diets formulated to contain sodium and chloride levels equivalent to a commercial control feed. Overall, the findings indicate that low‑level mussel meal inclusion, when mineral content is carefully controlled, does not negatively influence growth performance, hydration status, electrolyte balance, or health and welfare outcomes. These results support our hypothesis and suggest that mussel meal can be incorporated into broiler diets under appropriate formulation constraints.

Consistent with earlier work showing that mussel meal can maintain normal performance parameters in broilers (Waldenstedt and Jönsson, 2006), we observed no treatment effects on live weight or feed intake at any age. These findings provide further evidence that low inclusion levels of mussel meal do not affect the palatability or energy utilisation of commercial broiler diets. In contrast, the analysis of water intake revealed a more concerning picture. Although overall water intake increased with age as expected, the interaction between diet and age showed a modest but consistent increase in water consumption in the mussel‑fed group, amounting to 25.6 ml more water per bird per day by 32 days of age. This pattern did not translate into differences in growth or visible hydration‑related welfare problems; however, the higher water‑to‑feed ratio (1.86 vs. 1.75) suggests that birds compensated behaviourally for small differences in osmoregulatory demands. Nevertheless, such an increase in water consumption in a full scale commercial flock would be a significant increase that would need to be handled and offset by the ventilation system to avoid negative consequences on quality of the litter. In a flock of 20 000 birds, a typical broiler chicken flock size in Norway, this would add up to an extra consumption of 512 L of water per day by 32 days of age. In the winter season, when the ventilation is restricted to keep appropriate temperatures inside the barn, this offset strategy would not be so readily available and the results of such an increase in the water-to-feed ratio could have demonstratively serious consequences in litter quality, cleanliness, footpad dermatitis and gait score. In the present study, with only 18 birds per group, the litter quality was not obviously affected by diet treatment, remaining generally dry and loose. However, litter quality would be a very important indicator that would require monitoring in larger scale studies and in a commercial setting. It is important, however, to recognise that the method used for monitoring the water consumption in the present study was not very precise, as water containers had to be lifted in and out of the pens for measuring. In this process, some water may have spilled, giving imprecise results. Nevertheless, this does not explain the significant effect of diet treatment on these results, so they must still be considered in future research. Notably, however, plasma sodium and chloride concentrations did not differ between dietary groups, and all values remained within physiological ranges (Živkov Baloš et al., 2016). These findings contrast with the severe water imbalance and electrolyte dysregulation described in broilers fed high‑salt mussel meal diets (Tahamtani et al., 2026), reinforcing the critical importance of sodium and chloride control during feed formulation.

Additional physiological markers also did not detect any clear disturbance in the renal function. High salt concentration in the diet or water may lead to kidney damage and increased creatinine levels in the blood (Peng et al., 2022). In the present study, plasma creatinine and urea were below detection thresholds in nearly all samples, indicating no signs of renal overload or dehydration. Monitoring of electrolytes, particularly Na and Cl, and renal markers is important in this context because marine-derived feed ingredients have potential for a high mineral content, and presence of toxic elements (e.g., arsenic, lead, cadmium, mercury). The absence of kidney‑related changes in the present study is a key distinction from salt intoxication scenarios, where renal markers often rise sharply (Peng et al., 2022). Taken together, the physiological data show that although water intake increased slightly in mussel‑fed birds, electrolyte balance and renal function remained stable and within normal limits. Nevertheless, the sample size of the present study is limited and therefore, these results must be interpreted with care.

The health assessments, necropsy, mortality and welfare assessment provided further reassurance that the dietary treatment did not compromise health or comfort of the birds. No birds in either treatment group exhibited footpad dermatitis or bumblefoot, suggesting litter conditions and water distribution remained appropriate throughout the experiment. Although there was only a tendency for improved gait in mussel‑fed birds, this is noteworthy given that locomotor issues can arise when diets inadvertently affect hydration status or joint health. For example, high amounts of animal protein in feed can lead to lameness due swelling of the joints due to accumulation of uric acid (see review by Liu et al., 2023). Other indicators—including cleanliness, vent pasting, and valgus/varus deformities—showed no treatment effects. Minor findings, such as the presence of small comb pecking wounds only in control birds and the two mild hock burns observed in mussel‑fed birds, were sporadic and not consistent enough to suggest dietary causation. Overall, the welfare outcomes confirm that the 2 % mussel meal inclusion did not impair broiler comfort, integument condition, or leg health, as assessed by gait scoring. Likewise, the necropsy results reported the usual causes for culling in broiler chickens (Forseth et al., 2023, 2024), that is small, weak and lame birds, with sudden death syndrome, gizzard impaction, ascites and femoral head necrosis as the main findings and no difference between the diet treatments. Regarding mortality, it is important to note that the final mortality of 20-23 % is high relative to the average mortality at slaughter age of Norwegian broiler chicken flocks, of 2.72 % (Animalia, 2024). However, this is expected of such small scale controlled experiments where each individual bird represents a high percent of the flock and where researchers and veterinarians perform constant monitoring of the animals and cull those individuals that show less than ideal condition. The important take away from these data is, therefore, the lack of statistical difference between the groups, rather than the mortality itself. Nevertheless, the small scale of the present study is an important shortcoming of the study and cannot be overlooked. The results reported here must, therefore, be regarded with caution until they have been replicated in larger/full scale.

The results from the present study provide important context for ongoing efforts to identify sustainable alternative protein sources within the poultry sector. While most previous work has evaluated mussel meal as a replacement for fishmeal (Afrose et al., 2016; Jönsson et al., 2011; van der Heide et al., 2021a; Waldenstedt and Jönsson, 2006), our study demonstrates that mussel meal can also be incorporated into soy‑based commercial diets, broadening its potential applications. Because mussels require no feed inputs, contribute to water quality through filter‑feeding, and support circular bioeconomy models (Kotta et al., 2020; Suplicy, 2020; Timmermann et al., 2019), even low inclusion levels could play a meaningful role in reducing the environmental footprint of feed production systems. Extrapolating the inclusion of 2 % BMM used in the present study to the annual volume of chicken feed consumed in Norway results in a demand for 6000 tonnes of blue mussel protein meal which would replace imported protein feed ingredients such as soyabean meal. The absence of negative effects at a nutritionally modest but practically relevant inclusion rate supports the feasibility of commercial adoption. As such, mussel-derived ingredients hold potential both as a locally available protein source and as a contribution to circular bioeconomic systems (Timmermann et al., 2019). Indeed, several studies have investigated the effects of blue mussels on a human diet and found it to not cause adverse effects (Azad et al., 2023) and to aid in reducing our environmental footprint and potentially improve our nutritional intake and overall health (Yaghubi et al., 2021). There is also a large interest in the use of mussels and other protein alternatives for organic poultry production, as there is a limited availability in protein ingredients that meet organic and amino acid requirements (van der Heide et al., 2021b; Wilhelmsson et al., 2019). The mussels used in the present study were harvested from projects acting against the eutrophication of Danish waters and, as such, were not produced with the intent of human consumption. The use of blue mussel farming as a tool for improvement of water quality and removal of nutrients is well established as they filter seawater for plankton (Bayne, 1976; Maar et al., 2015). Due to this removal of nutrients such as nitrogen and phosphorus, mussel farming is a cost-effective measure for the mitigation of eutrophication of aquatic ecosystems, for example, in the Baltic Sea, and in Indonesia (Jusadi et al., 2021; Kotta et al., 2020; Lindahl et al., 2005; Schröder et al., 2014; Timmermann et al., 2019). Furthermore, it is also possible to utilise mussels produced for human consumption, without creating a direct food vs feed competition, by redirecting the discarded mussels (i.e. those too small and/or with broken shells) towards feed production instead of towards waste (Waldenstedt and Jönsson, 2006). Therefore, blue mussels farming has a large potential to supply a sustainable alternative protein ingredient for poultry feed production worldwide.

Nevertheless, the present study also highlights areas requiring future attention. The slight increase in water intake suggests that even when sodium and chloride levels are balanced, other components of mussel meal—such as residual inorganic content or osmolytes—may influence hydration behaviour. For example, the amino acid Glycine, found in high quantities in mussels (de Zwart et al., 2003), acts on osmoregulation, accumulating in the kidneys to protect against osmotic stress by increasing water retention in the cells (see review by Dobrijević et al., 2023). Therefore, an important remaining question is whether the small increase in water consumption observed in the present study, which did not otherwise affect the growth, health, or welfare of the chickens, would continue beyond day 32 of age and whether it would stay constant or continue to increase with time, and how this may affect ventilation rate and litter quality in commercial broiler settings. This is a relevant consideration for slower-growing hybrids, such as the Hubbard JA787 or the Ranger Gold hybrids, which are generally sent to slaughter between 13 and 38 days later than the Ross 308 hybrid used in the present study (Aviagen, 2018). In these cases, an increase in water consumption for a longer period might have adverse effects on the birds, such as reduced litter quality, increased footpad dermatitis and reduced cleanliness of the plumage (Manning et al., 2007) which were not observed here. Indeed, Wilhelmsson et al. (2019) found reduced litter quality and reduced plumage cleanliness in organic broilers feed a diet containing BMM at 6 weeks and 9 weeks of age, respectively compared to those fed a diet without BMM. Interestingly, in their study, 9 week old broilers fed a diet with mussel meal presented increased lameness compared to those fed a diet without mussel meal, which the authors attributed to the increased growth rate and body weight (Wilhelmsson et al., 2019). This was the opposite of the observed in the present study, where the Blue Mussel birds tended to have a better gait than the Control birds at 32 days of age and did not differ in body weight. In addition, mortality was reported to be higher in slower-growing broilers fed BMM compared to fast- and slower-growing birds fed diets without blue mussel meal (Wilhelmsson et al., 2019). Future work should therefore investigate the effects of low levels of BMM inclusion on longer production periods such as under organic production or flocks of slower-growing hybrids which will be feeding on the diet for a longer period of time.

In conclusion, the results of this study contribute to the growing literature on the effects of BMM as an alternative source of protein in broiler chicken feed. No adverse effects of an inclusion of 2 % BMM in broiler diets were detected on the growth and health of Ross 308 broiler chickens. Performance, welfare, and physiological measures remained within normal ranges, suggesting that BMM has potential as a sustainable feed ingredient in broiler production. However, care must be taken regarding the observed increase in water to feed consumption ratio and the potential consequences this could bring in full scale production. Further refinement of processing, mineral management, and inclusion strategies will help unlock the full value of mussel‑derived proteins within sustainable poultry nutrition frameworks.

## CRediT authorship contribution statement

**Fernanda Machado Tahamtani:** Writing – original draft, Methodology, Investigation, Formal analysis, Data curation, Conceptualization. **Hilde Faaland Schøyen:** Writing – review & editing, Project administration, Methodology, Funding acquisition, Conceptualization. **Käthe Kittelsen:** Writing – review & editing, Methodology, Investigation, Data curation, Conceptualization.

## Disclosures

We, the co-authors of the manuscript entitled “The effects of 2 % inclusion of Blue Mussel (Mytilus edulis) meal on the performance and clinical welfare of fast-growing broiler chickens” hereby declare the following conflicts of interest: Fiskå Mølle AS, a commercial feed mill, was involved in the production of the experimental feed used in this study. One of the co-authors of the present study is affiliated with Fiskå Mølle. While the company produced the feed and had access to the study design and the data collected, the authors affirm that the scientific integrity of the study has been maintained and that the conclusions presented are free from commercial influence.
